# Enhancing Feature Detection and Matching in Low-Pixel-Resolution Hyperspectral Images Using 3D Convolution-Based Siamese Networks

**DOI:** 10.3390/s23188004

**Published:** 2023-09-21

**Authors:** Chamika Janith Perera, Chinthaka Premachandra, Hiroharu Kawanaka

**Affiliations:** 1 Graduate School of Engineering, Mie University, Tsu 514-0102, Japan; kawanaka@elec.mie-u.ac.jp; 2 Department of Electrical Engineering and Computer Science, Graduate School of Engineering and Science, Shibaura Institute of Technology, Tokyo 135-8548, Japan; chintaka@sic.shibaura-it.ac.jp

**Keywords:** hyperspectral imaging, feature matching, 3D convolution Siamese network

## Abstract

Today, hyperspectral imaging plays an integral part in the remote sensing and precision agriculture field. Identifying the matching key points between hyperspectral images is an important step in tasks such as image registration, localization, object recognition, and object tracking. Low-pixel resolution hyperspectral imaging is a recent introduction to the field, bringing benefits such as lower cost and form factor compared to traditional systems. However, the use of limited pixel resolution challenges even state-of-the-art feature detection and matching methods, leading to difficulties in generating robust feature matches for images with repeated textures, low textures, low sharpness, and low contrast. Moreover, the use of narrower optics in these cameras adds to the challenges during the feature-matching stage, particularly for images captured during low-altitude flight missions. In order to enhance the robustness of feature detection and matching in low pixel resolution images, in this study we propose a novel approach utilizing 3D Convolution-based Siamese networks. Compared to state-of-the-art methods, this approach takes advantage of all the spectral information available in hyperspectral imaging in order to filter out incorrect matches and produce a robust set of matches. The proposed method initially generates feature matches through a combination of Phase Stretch Transformation-based edge detection and SIFT features. Subsequently, a 3D Convolution-based Siamese network is utilized to filter out inaccurate matches, producing a highly accurate set of feature matches. Evaluation of the proposed method demonstrates its superiority over state-of-the-art approaches in cases where they fail to produce feature matches. Additionally, it competes effectively with the other evaluated methods when generating feature matches in low-pixel resolution hyperspectral images. This research contributes to the advancement of low pixel resolution hyperspectral imaging techniques, and we believe it can specifically aid in mosaic generation of low pixel resolution hyperspectral images.

## 1. Introduction

Hyperspectral imaging is becoming one of the main technologies driving the remote sensing research field thanks to its ability to see beyond the visible spectrum and provide the information needed in decision-making. A hyperspectral image is a 3D representation of the scene it captured, with two spatial dimensions and one spectral dimension. The spectral dimension usually consists of bands both within and outside the visible spectrum. Typically, the total number of bands can range from tens to hundreds. Each band contains information from a narrow region of the electromagnetic spectrum. In the aerial hyperspectral domain, this is the information reflected by different materials in the terrain. Because the electromagnetic spectrum reflected from each material is different, depending on the material properties and material current status a unique spectrum can be captured using hyperspectral images. This unique spectrum, known as the spectral signature, serves as the foundation for hyperspectral imaging in remote sensing. Two main image capture methods are associated with aerial hyperspectral imaging, namely, push-broom and snapshot acquisition. In push-broom acquisition, the sensor consists of an array of single pixels and the mounted platform is moved across the target area that needs to be captured. In snapshot imaging, target areas are captured as a cube containing both spectral and spatial information.

Feature detection and matching between hyperspectral images is recognized as a critical step in various application areas of hyperspectral imaging. One prominent application is the image stitching process. By stitching the captured image into one composite image, the user can interpret the captured data relative to the total captured area, rather than interpreting it on a per-image basis. This is particularly important in snapshot images. Due to hardware and software limitations, these images are relatively small in terms of their pixel dimensions when processing snapshot sensor data. Hence, a narrower field is captured compared to push-broom methods. Change detection over a time period [1] is another application in which feature matching is important. When there is a temporal difference between the captured images, the images need to be aligned based on common features in order to detect the change. Similar to change detection, object detection and tracking [2] is another area in which feature matching is important. Apart from these, anomaly detection can be identified as an area in which feature matching is currently being utilized.

The main entry barrier to the hyperspectral research field is the cost associated with camera systems. However, recently introduced low-resolution hyperspectral cameras try to address this drawback by bringing the cost down to a comparatively low price point. Furthermore, the form factor of these cameras is being reduced to facilitate the process of incorporating the cameras into low-cost aerial platforms. At the same time, this reduction in cost and form factor comes with the main trade-off of lower pixel resolution. In this paper, we focus on the problems associated with feature matching of these low pixel resolutions and propose a novel feature matching method based on incorporating both the spectral and spatial information that is present in hyperspectral images.

Feature matching is the process of identifying common features between images in order to understand the relationship between images or areas of interest in images. These matched features are used in different application areas, such as orthomosaic generation, image-based localization, object recognition, and tracking. However, this step faces challenges in low-resolution hyperspectral image matching due to factors such as low contrast, repeating patterns, and images with low texture. This problem becomes more challenging when images are captured using low-altitude flying conditions.

Our previous research introduced this feature matching problem and elaborated on the low performance of traditional feature matching methods using selected image samples [3]. Furthermore, we proposed the recently introduced LoFTR, a detector-free transformer-based feature matching method, as a solution to be used with low-resolution hyperspectral images [4]. However, even though the proposed adaptation of the LoFTR method was successful at identifying feature matches needed in the transformation calculation process, our experiments revealed cases of image pairs that produced a low number of feature matches or inaccurate feature matches. Traditional feature matching in hyperspectral imaging is mostly performed by selecting a specific spectral band from the images, then performing feature detection and matching with respect to the selected spectral band. Almost no methods have used the information present in different spectral bands in the feature matching process. Therefore, we hypothesize that the use of both spatial and spectral information from hyperspectral image pairs will result in highly accurate feature matches.

In this research paper, we propose a novel method to increase the robustness of feature match detection by incorporating both spectral and spatial information. In order to achieve this, Phase Stretch Transformation is used to generate a set of initial feature matches from the matched image pairs, then a Siamese network based on a 3D Convolution Neural Network is utilized to filter out inaccurate filter matches by thresholding the Euclidean distance of the network output embeddings. The main contributions of this research paper are as follows:

- A feature match generation method using phase stretch transformation-based edge maps.
- Training, evaluation, and application of a Siamese network based on a 3D convolution neural network for feature filtering.
- Evaluation of the proposed method with the state-of-the-art methods.

The results of the proposed method suggest its ability to generate similar outcomes to traditional methods in cases where these have been successful in producing high quality matches. In the instances where traditional methods fail or produce less accurate feature matches, the proposed method shows improved performance. In the forthcoming sections of this paper, we discuss the approaches followed, the experiments performed, and the results obtained. Specifically, the next section discusses the related work in the areas that this research focuses on. Section 2 presents the methodology that we followed to generate the feature matches and filter them out in order to obtain a set of filtered feature matches with high accuracy. Section 3 discusses the experiments performed to evaluate the proposed method and discusses the obtained results. Finally, Section 4 concludes the research and discusses future work that we suggest to increase the robustness of the proposed framework.

## 2. Related Work

### 2.1. Hyperspectral Imaging in Remote Sensing

The hyperspectral imaging technique was first introduced in the 1980s; Goetz et al. published the first paper on hyperspectral imaging, titled “Imaging Spectrometry for Earth Remote Sensing” [5], describing the process as acquiring images simultaneously in 100 to 200 contiguous spectral bands. It was first developed as airborne and spaceborne sensors by the National Aeronautics and Space Administration (NASA) to observe the earth’s surface. With recent advancements in technology, sensors have become smaller, making them more accessible and mountable on different platforms, including manned aircraft, Unmanned Aerial Vehicles (UAVs), and laboratory-based acquisition systems. In the remote sensing domain, being able to mount these acquisition systems on UAVs has opened up whole new streams of research, in the agriculture remote sensing domain [6,7,8,9] as well as in such application areas forestry [10,11], environmental monitoring [12,13], security [14], and geology [15].

From the literature, it can be identified that most of the hyperspectral cameras used in UAV-mounted hyperspectral image acquisition systems involve either line scanning technology or snapshot acquisition technology [16]. Line scanning sensors are equipped with an array of pixels; each pixel captures a spectral signature, and the acquisition platform needs to be moved in order to capture the target area. These cameras require advanced postprocessing to remove the geometric distortions that occur when the line-by-line image is stitched into a single image. Snapshot cameras, on the other hand, capture information from the available field of view in a single instance. A recent addition to the field is low-resolution snapshot sensors, which attempt to address the high entry cost barrier of hyperspectral imaging. Imaging cameras such as the Cubert Ultris 5 [17], with a spatial resolution of 275 × 290 pixels, and the Ximea snapshot cameras [18], with a resolution of approximately 409 × 217 pixels, are two such systems. However, their low pixel resolution poses a new set of challenges to overcome if the users are to successfully adapt these systems into the decision-making process. One such challenge involves the difficulty of detecting a robust set of feature matches from overlapping regions in image pairs.

### 2.2. Dimensionality Reduction of Hyperspectral Images

Dimensionality reduction is a critical step in the neural network training process. It makes the feature extraction process more efficient for the neural network by allowing it to focus on relevant features and discard less valuable features. In addition, it increases computational efficiency in both training and inference. Regularization to prevent overfitting is another advantage of dimensional reduction. This is critically important in hyperspectral imaging due to the high number of spectral bands. Dimensionality reduction can be divided into two main methods, namely, linear and nonlinear. Principal Component Analysis (PCA) [19] is one of the most commonly used linear methods, while Linear Discriminant Analysis (LDA) [20] and Independent Component Analysis (ICA) [21] are among the other commonly used linear methods of dimensionality reduction. Nonlinear dimensionality reduction methods include Kernal PCA [19], Local Linear Embedding (LLE) [22], and Laplacian Eigenmaps [23].

With advancements in machine learning, autoencoder-based dimensionality reduction methods are being used successfully in hyperspectral image processing tasks [24,25,26,27]. Autoencoders are a nonlinear dimensionality reduction technique that can represent the data in a lower dimensional space while preserving the important features. This involves an unsupervised learning neural network consisting of an encoder that maps the higher dimensions into a lower dimensional space and a decoder that reconstructs the input data from the lower dimensional space data created by the encoder. With an appropriate amount of training data, the literature indicates that autoencoders demonstrate superior performance in comparison with other methods such as PCA, kernel PCA, nonlinear PCA, and ICA [28,29].

### 2.3. Siamese Networks

Siamese networks were first introduced in 1993 in the paper titled “Signature verification using a Siamese time delay neural network” [30]. The basic architecture of a Siamese network consists of two or three identical neural networks that share the same weights. The networks are trained with matching and non-matching image pairs, resulting in a meaningful representation of the input data being learned in such a way that similar inputs are mapped more closely than the dissimilar inputs in the learned feature space. This allows Siamese networks to be used in tasks such as similarity-based classification and verification [31]. A twin Siamese network is trained with similar and dissimilar image pairs, while a triplet network is trained with an anchor image, a similar image, and a dissimilar image. In the training process, these two types of networks use two different loss functions: a contrastive loss function in the twin network architecture, and a triplet loss function in the triplet architecture. Applications of Siamese networks include object tracking [32], face recognition [33], and signature verification [34]. In the hyperspectral image domain, research can be found in the application areas of image classification [35] and object/target detection [36,37]. However, our literature survey did not unveil any research articles which have investigated the possibility of using a Siamese network for feature matching in hyperspectral imaging, which opens up space for new research.

### 2.4. Feature Matching for Hyperspectral Imaging

The traditional approach to detecting and matching features in hyperspectral images is to select a specific band from the available band set and then apply normal feature matching methods designed for grayscale images. These feature matching methods can be divided into two main subsections based on their feature detection method, namely, learning-based feature detection methods and methods which do not incorporate learning. Traditional methods, such as frequency domain methods [38], spatial domain methods [39], optical flow methods [40], Scale-Invariant Feature Transform (SIFT) [39], Speeded-Up Robust Features (SURF) [41], Harris corner detection [42], Features from Accelerated Segment Test (FAST) [43], Binary Robust Invariant Scalable Keypoints (BRISK) [44] and Oriented FAST and Rotated BRIEF (ORB) [45], fall under the heading of non-learning-based methods. With the progress of deep learning technologies, more sophisticated methods of feature detection and matching have been introduced to address drawbacks in non-learning-based methods. These drawbacks include poor performance on low texture images, image pairs with varying illumination, images with repetitive patterns, and images with motion blur.

Learning-based feature detection and matching methods have been proposed in multiple different research articles, and include Learned Invariant Feature Transform (LIFT) [46], MagicPoint [47], SuperPoint [48], SuperGlue [49], NcNet [50], sparse NcNet [51], and DualRC-Net [52]. Here, we focus only on the most recent additions to the feature detection and matching space and algorithms that are readily available for public use; local Feature matching with TRansformers (LoFTR) [4], introduced in 2021 and local feature matching at light speed (LightGlue) [53], introduced in 2023, are two of the latest methods available in the public domain for feature detection and matching. The LoFTR method proposes a transformer-based architecture with self- and cross-attention layers to obtain feature matches; the authors’ performance evaluation stated that the proposed method outperforms state-of-the-art methods by a considerable margin. LightGlue is a feature matcher that can be used with a variety of keypoint detectors. It uses a transformer-based backbone similar to LoFTR, with self- and cross-attention layers in the LightGlue detector and matcher. However, the authors proposed a different strategy to derive the exact matches from initial predictions using a pruning layer and matching layer. Their evaluation stated that this method achieves a relatively high level of accuracy compared to state-of-the-art methods while being far more efficient in terms of speed.

Moving on to the literature on hyperspectral feature matching, in image stitching research Yi et al. [54] and Peng et al. [55] have discussed using SIFT as a feature matcher. Mo et al. [56] and Zhang [57] discussed using SuperPoint [48] and SuperGlue [49] for the feature detection and matching step. Fang et al. [58] discussed the use of the Spline Sparse Bundle Adjustment (SSBA) method for image registration tasks. KAZE features, a multiscale 2D feature detection and description algorithm [59], were incorporated by Ordonex et al. [60] for the task of aligning hyperspectral images. Another new approach called the Unified Model of Spectral value and Gradient Change Information SIFT (UMSGC SIFT) was proposed by Li et al. [61], consisting of a spatial and spectral SIFT algorithm for hyperspectral image matching tasks. However, none of these papers has discusses low-resolution hyperspectral images or addressed the associated challenges.

## 3. Methodology

**Figure 1:**
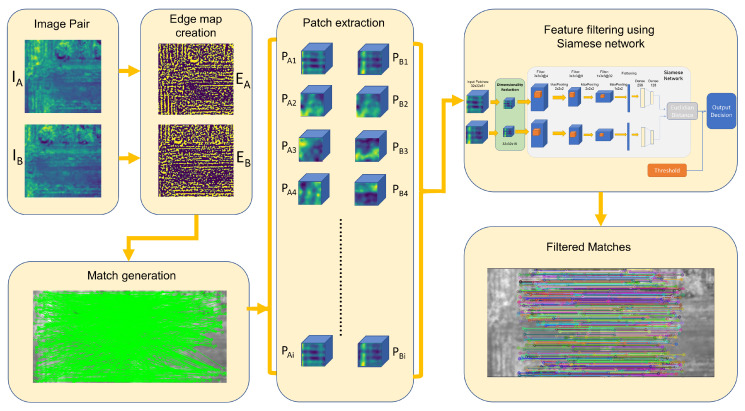
Overview of the proposed method.

Figure 1 presents an overview of the proposed method. Four main steps are performed in order to generate a set of highly accurate filter matches from a given image pair $I_{A}$ and $I_{B}$. The first step of the proposed method involves selecting a spectral band, extracting edges from each image pair, and generating a binary edge map using Phase Stretch Transformation ($E_{A}$ and $E_{B}$). In the next step, SIFT feature detection is carried out for each edge map and a brute force matcher is used to obtain a set of matches from the two edge map images. In the next step, 32 by 32 patches around the identified matched keypoints are extracted from each selected patch ({$P_{A1}$, $P_{A2}$, *…*, $P_{Ai}$} and {$P_{B1}$, $P_{B2}$, *…*, $P_{Bi}$}) along with all of the spectral band information. In the final step, a 3D convolution-based Siamese network is utilized to filter out inaccurate feature matches from the detected list of feature matches. The following subsections discuss each of these processes in detail.

### 3.1. Data Acquisition and Preprocessing

**Figure 2:**
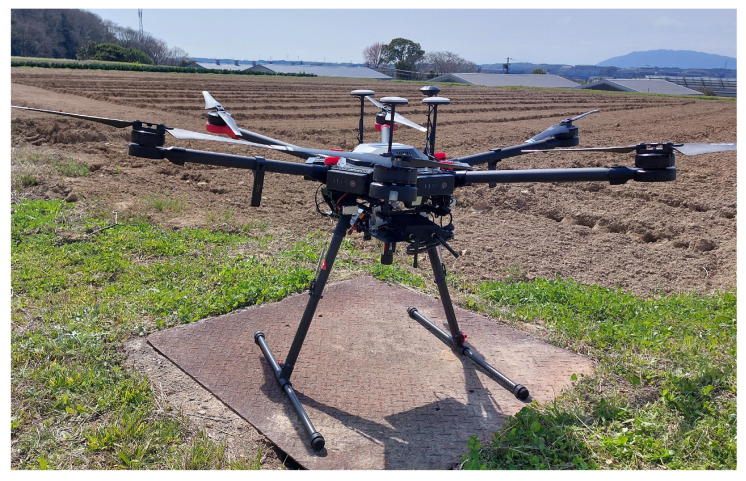
The DJI M600 Pro drone with mounted camera.

All of the hyperspectral images used in this experiment were acquired using the Cubert Ultris 5 hyperspectral camera [17]. It has a resolution of 290 pixels by 275 pixels, 51 spectral bands sampled from 450 nm to 850 nm, and a spectral resolution of 8 nm. With dimensions of 29 mm by 29 mm by 40 mm and a weight of 120 g, it represents an ideal solution for mounting on a wide range of UAVs. Together with the provided single-board computer, triggering circuit, and wiring-mounting hardware, the total acquisition system weighs under 800 g. A DJI M600 pro drone was used for the data acquisition sessions, and the provided SDK was used to convert the captured irradiance images to radiance. Figure 2 depicts the data acquisition platform. More information about the setup and the initial data conversion process can be found in the paper [62].

Image acquisition was carried out using DJI Ground Station Pro software v2.0.17, with “hover and shoot” mode used to trigger the hyperspectral camera. Front and side overlaps between images of 80% were selected due to the accuracy limitations of the traditional GPS systems in order to ensure a sufficient common area between two images, as a high overlap percentage allows for better detection of feature matches. The selected fields for data acquisition contained a mix of the following: freshly ploughed soil, a mix of weeds and soil, watered paddy fields, asphalt roads, fully grown weeds, and orange plants. These image scenes were selected in order to demonstrate the feature detection limitations that occur in the agriculture plots, such as vegetation in its early stages when a large percentage of soil remains visible.

### 3.2. PST-Based Feature Match Generation

Training a Siamese network to distinguish matches from non-matches using a set of potential feature matches is the primary approach used in this paper to obtain a set of robust filter matches. The most rudimentary approach is to select a region of pixels from the first image and then obtain all the possible combinations from the second image with the same region size. This is repeated until the full image area in the first image is covered. However, this is a resource-intensive task; another approach is to convert the image into a different domain and then find features from it. After investigating several approaches, such as hyperspectral band fusion and image enhancement, we determined that generating edge maps from the hyperspectral image and then finding features in the edge map resulted in a dataset that was sufficiently accurate to be adequately filtered by the Siamese network.

Phase Stretch Transformation (PST) is a computationally efficient edge and texture detection method that promises exceptional performance on visually impaired images. It was first introduced by Asghari et al. in the papers [63,64] based on the concept of photonic time stretch [65]. In the search for a suitable edge map creator, [66] evaluated phase stretch transformation-based edge detection in comparison to several different popular edge detection algorithms (canny edge detection [67], Sobel edge detection [68], and Laplacian of Gaussian (LoG) edge detection [69]), finding that edge detection with PST resulted in superior performance. We did not investigate learning-based edge detection methods, which was due to several reasons, including the trained algorithm not being available, the trained data size being different from the size of the low-resolution hyperspectral images, and the high computational burden.

At first, one band from each hyperspectral image was selected to apply the PST edge detector. Then, each image was passed through the PST algorithm using the Python library [66]. There are five parameters in the algorithm, which the user is able to tune: the phase strength, warp strength, standard deviation (sigma) of the Gaussian low-pass filter, lower and upper thresholds, and a boolean for binary/analog edge detection. Phase strength and warp strength were selected as 0.2 and 50, respectively, while the sigma of the low pass filter was selected as 0.15. The threshold range was from 0 to 0.65, and the binary mask was taken out as the edge map. Next, the converted edge image was processed through the SIFT feature detector and KNN matcher, using opencv to obtain a list of matches for the image pair. The sensitivity of the SIFT detector was increased by changing the contrast threshold, sigma, and noctaveLayers parameters. In our experiment, the minimum contrast required for keypoint detection was set to zero and the sigma of the Gaussian smoothing kernel was set to 1. The number of octaves used to detect keypoints was set to 8.

**Figure 3:**
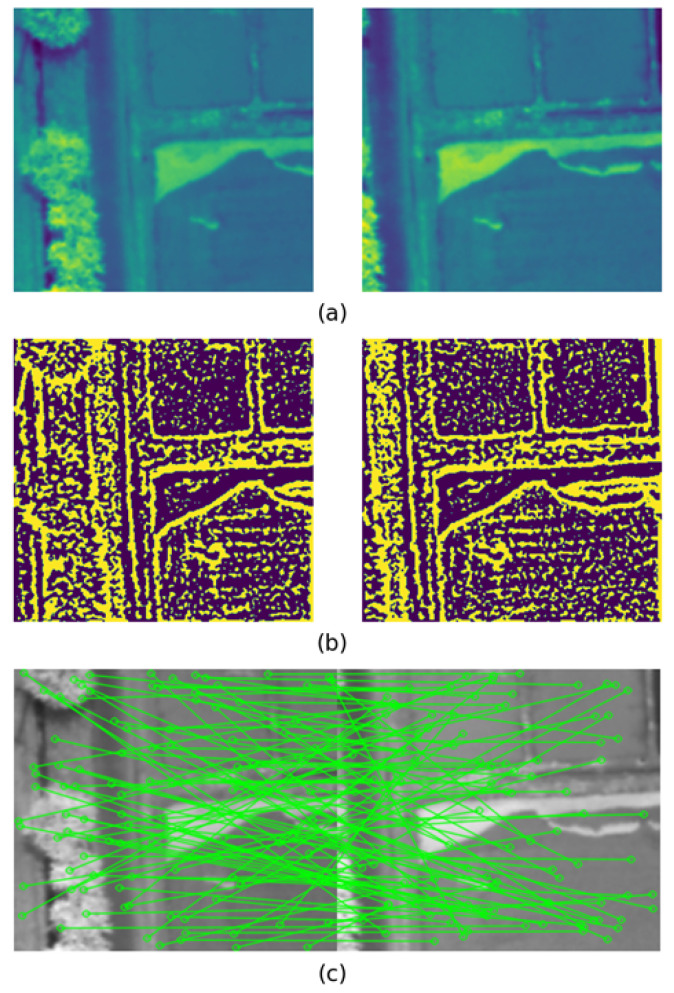
Intermediate results of feature match generation method: (**a**) selected image pair from the 750 nm band, (**b**) PST edge map, (**c**) random sample 100 detected matches.

By adjusting the SIFT detector’s sensitivity, more than 10,000 feature matches were generated, providing ample data for the Siamese network to utilize. The selection of the spectral band used to perform edge detection was partially important when generating the feature matches in this step. Even though the generated feature matches remained high no matter the selected band, and each band produced enough correct feature matches to calculate the homography in the majority of cases, we identified that selecting a spectral band from 600 nm to 800 nm resulted in highly accurate matches. Further investigation into the specific results is presented in the Section 4.1. Figure 3a depicts the original images, Figure 3b the edge map created using the PST method, and Figure 3c 100 of the randomly selected feature matches of the selected image pair. This process was followed by extracting patches with a pixel size of 32 × 32 while keeping the matched keypoints as the center of the patch. Each patch was extracted along with all the spectral information in the patch area, which resulted in two 32 × 32 × 51 image patches for each matched keypoint shown in the first and second images.

### 3.3. Dimensionality Reduction Using Autoencoder

Training a neural network with the obtained 32 × 32 pixel patches extracted with the 51 bands is not a recommended procedure to follow due to the challenges associated with high dimensionality, such as the curse of dimensionality [70]. Hence, a simple autoencoder-based dimensionality reduction network was implemented in this research to reduce the dimensions of the hyperspectral image from 51 bands to 16 bands. The decision to use autoencoder-based dimensionality reduction was based on the execution time. Even though training the autoencoder is computationally expensive, the time to reach inference with the trained model is much faster than more traditional methods such as Principal Component Analysis.

Dimensionality reduction was achieved using a feed-forward neural network-based encoder and decoder, with three hidden layers in each. Instead of representing the whole patch in a lower-dimensional space, a 1D autoencoder was trained to reduce the dimensions of each spectral signature at each pixel; hence, the size of the input to the autoencoder is (1, 51). Each hidden layer is followed by a batch normalizing layer and a leaky ReLU layer. After the training process, the encoder portion of the autoencoder reduces the 51 dimensions to 16 at each pixel of the 32 × 32 patches. The regenerated pixel spectra from the autoencoder showed a high correlation with the original data, solidifying the selection of the autoencoder approach.

### 3.4. Feature Filtering Using a 3D Convolutional Siamese Network

**Figure 4:**
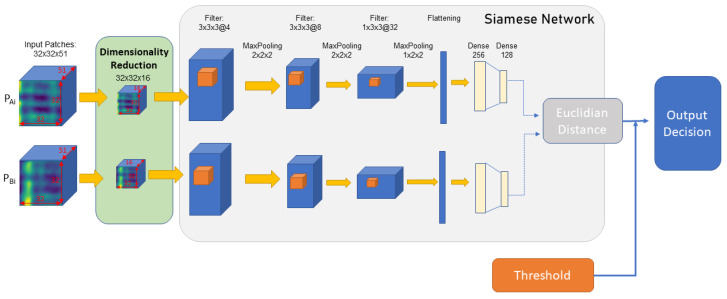
Proposed network architecture.

The generated feature matches consisted of two subsets, correct matches, and incorrect matches, with the Siamese network used to distinguish matches from non-matches. In order to utilize both the spectral information and spatial information during the filtering process, a 3D convolution-based Siamese network is proposed in this research. Figure 4 depicts the architecture of the proposed Siamese model. The network was trained on matched and non-matched features, with an input size of 32 × 32 × 16 in each patch.

#### 3.4.1. Network Architecture

The network consists of three 3D convolution layers, with 3D convolution kernel sizes of 3 × 3 × 3 for the first two layers and 1 × 3 × 3 for each successive layer. After each convolution layer, batch normalization is carried out and a LeakyReLU function with a negative slope set to 0.2 is used as the activation function. After each activation layer, three maxpooling layers are used in the size of 2 × 2 × 2 for the first two layers and 1 × 2 × 2 for the final maxpooling layer. After this, the output is flattened and input to a dense network with two layers having sizes of 256 and 128, respectively. Finally, the Euclidian distance is calculated from the output of each network. In order to increase the distance of the output embeddings for non-matched images and decrease the distance of output embeddings for matched images, a contrastive loss function is adopted during the training process. The contrastive loss function is designed to penalize the network when the two inputs are the same and reward it when they are different. This proposed network shape was obtained after experimenting with several shapes and sizes to find the one that provided the best accuracy.

The contrastive loss function is calculated as follows. Considering that $z_{1}$ and $z_{2}$ are embeddings of two patches in an embedding space, *y* is the binary label indicating whether or not the pair of patches is a match, and margin represents a hyperparameter that defines the minimum desired separation between similar and dissimilar points in the embedding space, the calculation of the contrastive loss function is performed as follows.

First, compute the Euclidean distance (L2 distance) between the two embeddings by calculating the element-wise difference between $z_{1}$ and $z_{2}$. Then, calculate the squared L2 distance for each pair of embeddings. Finally, calculate the Euclidean distance by taking the square root of the squared distances represented in Equations (1) and (2).

(1) $$difference=z_{1}-z_{2}$$

(2) $$Euclidean\_Distance=\sqrt{\sum_{i=1}^{n}{\left({\mathrm{difference}}_{i}\right)}^{2}}$$

Then, calculate the difference between the margin and the calculated distance. The negative values are clamped to ensure that negative values are set to zero, as indicated in Equation (3). This ensures that dissimilar points are only penalized if they are closer than the desired margin. Finally, the loss for both patches is calculated using Equation (4).

(3) $$negative\_distance=min(max\left(\left(margin-Euclidean\_Distance\right),0.0\right),max\_value_{i})$$

(4) $$\mathrm{loss}=\frac{y\cdot \mathrm{distance\_squared}+\left(1-y\right)\cdot {\mathrm{negative\_distance}}^{2}}{2.0}$$

**Figure 5:**
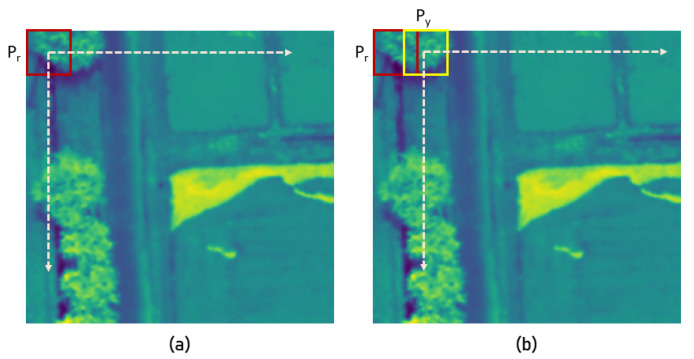
Training data creation process: red square = $P_{r}$ window, yellow square = $P_{y}$ window. (**a**) matches and (**b**) non-matches.

The training process was carried out using a dataset created by moving a 32 × 32 pixel window along one image. As Figure 5a demonstrates, matches in the dataset were created by duplicating the same image patch while moving the $P_{r}$ window with a stride of 5 pixels. This 32 × 32 window was then moved until the entire image was covered, and duplicated patches were saved as matches. For non-matches, the $P_{r}$ window was kept stationary while moving the $P_{y}$ window by 5 pixel strides to cover the entire image. Each non-match consisted of a patch $P_{r}$ and patch $P_{y}$. When the $P_{y}$ window covered the whole image, the $P_{r}$ window was moved by a 5 pixel stride; this movement of $P_{y}$ was repeated in such a way that duplicates were avoided. This combination of movements resulted in 2548 matches and 1,685,502 non-matches from one image. However, non-matches were randomly dropped, and only 5000 non-matches were recorded from one image. In all, 300 hyperspectral images were selected from different acquisition sessions to create the data samples for training and validation.

#### 3.4.2. Training Dataset Creation and Training Process

**Figure 6:**
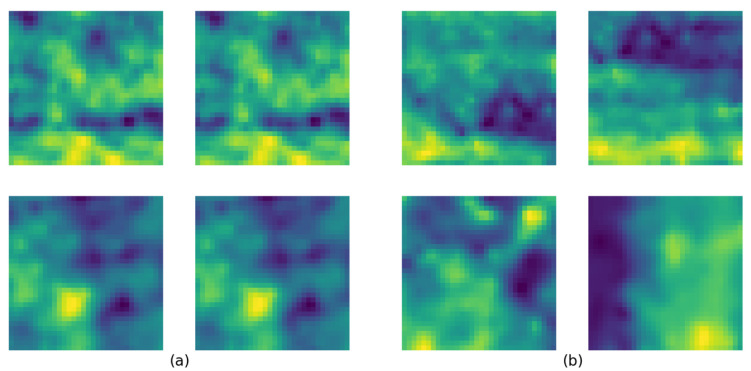
Four samples from the created dataset: (**a**) matches and (**b**) non-matches.

**Figure 7:**
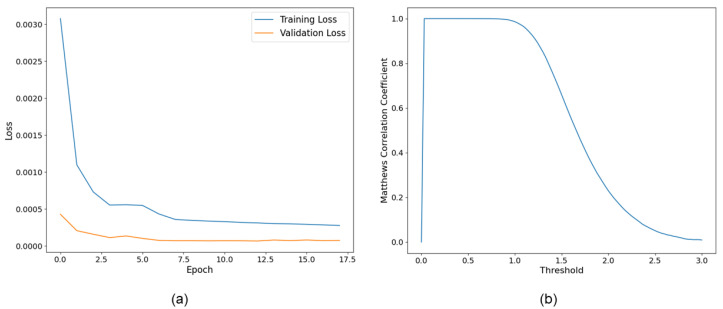
(**a**) Training and validation loss curve and (**b**) Matthews Correlation Coefficient curve.

Out of the created dataset, 7,000,000 matches and non-matches from each class were randomly selected for the training, validation, and test process. Figure 6a depicts two matched samples from the dataset and Figure 6b depicts non-matched samples. Selected data were divided into training, validation, and testing sets in a ratio of 0.8, 0.1, and 0.1. Model training was stopped at epoch 18 under the criteria that five consecutive validation losses did not show any improvement. The model was trained at a learning rate of 0.0001 using Adam optimization [71]. Furthermore, dynamic mini-batch sizing was used in the training process, where the batch size was increased over the first five epochs from 256 to 2048. Studies suggest that incorporating this approach in large-scale models provides the advantages of fast convergence and a regularization effect while saving memory and computational resources [72,73]. Although the proposed model has only 202,568 tunable parameters, which is relatively small compared to many other models, we believe that this aids in its fast convergence. Figure 7a presents the training and validation loss values for the model.

After the training process, inference was carried out to obtain the prediction by calculating the same Euclidian distance and then thresholding the distance. Figure 7b presents the Matthews Correlation Coefficient curve for the test dataset. The Matthews Correlation Coefficient (MCC) is a performance metric for binary classification tasks that takes into account true positives, true negatives, false positives, and false negatives. The score falls between [−1, 1], where 1 represents a perfect prediction, 0 represents a random prediction, and −1 represents total disagreement between prediction and observation. The MCC curve is obtained by changing the threshold value and calculating the respective MCC value. In Figure 7b, the x-axis represents the threshold values considered from 0 to 3 and the y-axis presents the MCC value obtained at each threshold. The curve indicates that threshold values less than 0.9 produce near-perfect results for the test set. However, when the trained model was applied to adjacent image pairs, thresholds above 0.6 indicate a considerable number of incorrect matches. This is due to the slight difference between the trained data and real data. The trained data were identical matches from the same images; however, when the trained algorithm was applied to adjacent images, slight illumination and perspective changes occurred due to changes in the image acquisition location. The incorporated method for the generation of the data set completely removes the labor intensity required if the dataset is generated with adjacent images with manual labeling. We believe that the features learned from the dataset created with the proposed method were successful at teaching relevant features to the CNN, which we demonstrate in the results section.

### 3.5. Evaluation Procedure

In order to evaluate the proposed method, several hyperspectral image pairs were selected, demonstrating the superior performance of the proposed model as well as instances in which it does not perform well. The 762 nm (38th) spectral band was selected from each of the image pairs, then the edge image was created as described in Section 3.2. Next, a set of feature matches was obtained using SIFT detectors, as described in the same section. Patches with a size of 32 × 32 were extracted in pairs for each match, and the dimensions were reduced from 51 to 16 using the encoder. Each pair of patches were then processed through the Siamese network to obtain the Euclidian distance. A threshold value was selected for binary classification. Then, the set of filtered feature matches was used to evaluate the proposed method with both traditional methods and state-of-the-art methods. Several evaluation metrics were used to compare the methods. The inlier percentage was obtained by calculating the homography with RANSAC. Furthermore, after extracting a common region from the two overlapping regions following the homography transformation, the Structural Similarity Index Metric (SSIM) [74] and correlation coefficient for the two regions were calculated. The SSIM is derived by calculating the three subcomponents of luminance, contrast, and structure; hence, the SSIM is correlated with the accuracy of the identified feature matches. On the other hand, the correlation coefficient measures the linear relationship between image pixels in the two regions. Coefficient values range from −1 to 1, with −1 indicating a perfect negative linear correlation and 1 indicating a perfect positive correlation. However, this metric does not consider the perceptual qualities of the two regions.

In order to compare the proposed method, the aforementioned LoFTR and LightGlue methods were selected. LightGlue was utilized along with the DISK feature detector [75] during the experiments based on the superior performance observed for this combination of feature detector and matcher. In addition, a combination of the Good Features to Track (GFTT) detector [76] + OpenGlue [77] and a combination of the KeyNet keypoint detector [78] + OpenGlue were utilized as state-of-the-art methods with which to compare the performance of our proposed method. A second-nearest neighbor matcher (SNN) was utilized to filter the non-matches from the matches in each of the aforementioned detectors and descriptors. OpenGlue uses a convolutional neural network to produce descriptors for keypoints identified using GFTT and KeyNet. The methods used for comparison were selected after an initial evaluation in which they produced comparable results with the LoFTR and LightGlue algorithms.

## 4. Results and Discussion

Our results and the associated discussion are presented in two subsections. First, we discuss the use of the spectral band in PST-based feature match generation and how selecting a particular band affects the performance of the proposed method. Next, the performance of the proposed method is evaluated using selected image pairs from several different datasets captured in different locations.

### 4.1. Selection of the Spectral Band

**Figure 8:**
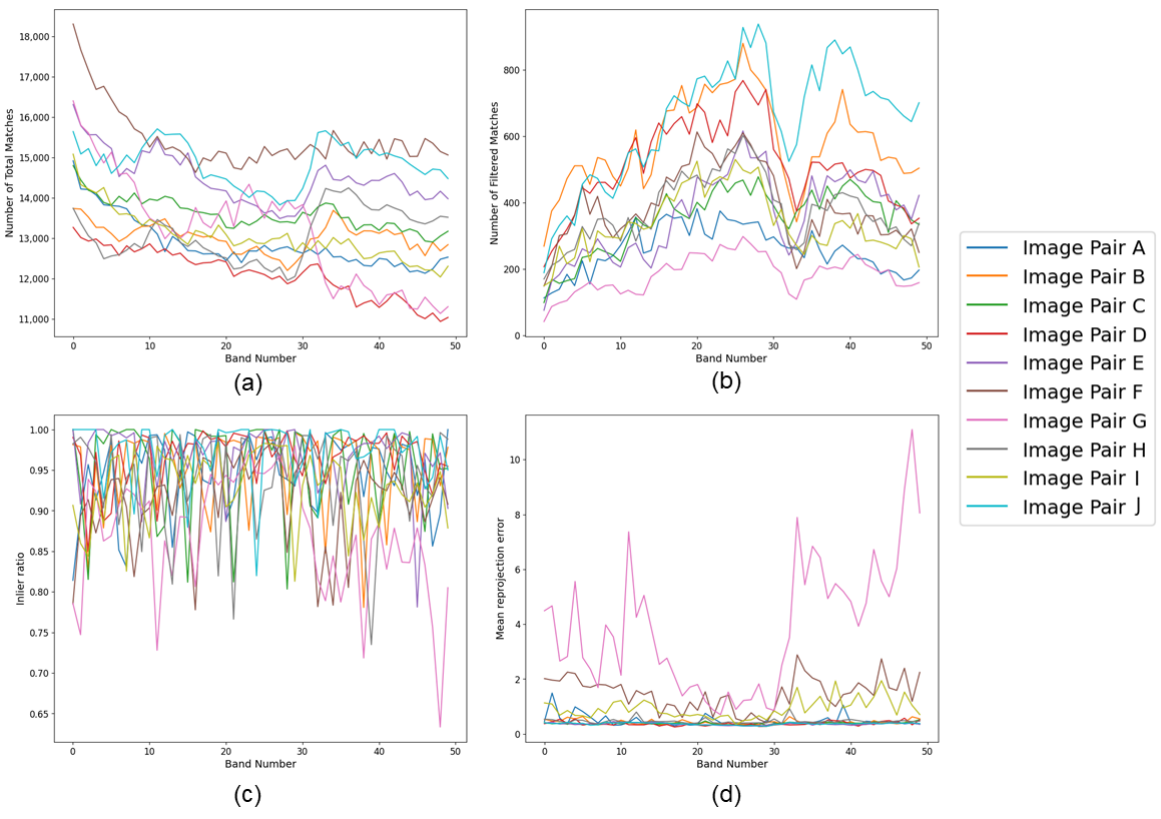
Evaluation of band selection for initial feature matching for ten image pairs: (**a**) total matches produced for each band, (**b**) total matches produced by the proposed method, (**c**) inlier ratio for each band, (**d**) mean reprojection error for each band.

As mentioned in Section 3.2, we identified that the performance in the 600–800 nm range (band 18 to band 42) was comparatively better than that in the other spectral bands. Figure 8 presents graphs obtained to evaluate this statement. Ten image pairs were randomly selected and feature matching was carried out for each band of the hyperspectral image pair. Figure 8a presents all of the matches obtained for each image pair at each spectral band, while Figure 8b presents the filtered features obtained from the proposed Siamese model. The derived feature matches were then used to calculate the homography between the two images at each band, and the inlier ratio and mean reprojection ratio were calculated for each band. Figure 8c,d presents the respective plots. These graphs highlight that a high number of matches identified in the initial few bands does not necessarily translate into accurate match predictions. This is due to the fact that the contrast between pixels in the initial bands is lower, which produces noise rather than accurate matches when the image is subjected to PST-based edge detection. Bands 32 and 33 suffer from the same situation, with a drop in the filtered features. In terms of inliers, most of the bands from all image pairs were able to produce more than 80% inliers; however, image pair G had fluctuating performance, producing a respectable inlier ratio and a reprojection error between bands 20 and 30 (618–706 nm). These results suggest that in order to obtain the maximum performance with the proposed method it is necessary to consider the contrast between the pixels within each band. Notably, when incorporating edge-based feature detection in an application area other than agricultural plots, such as biomedical hyperspectral imaging it is necessary to consider the most band suitable for the specific application.

### 4.2. Evaluation of the Proposed Method

**Figure 9:**
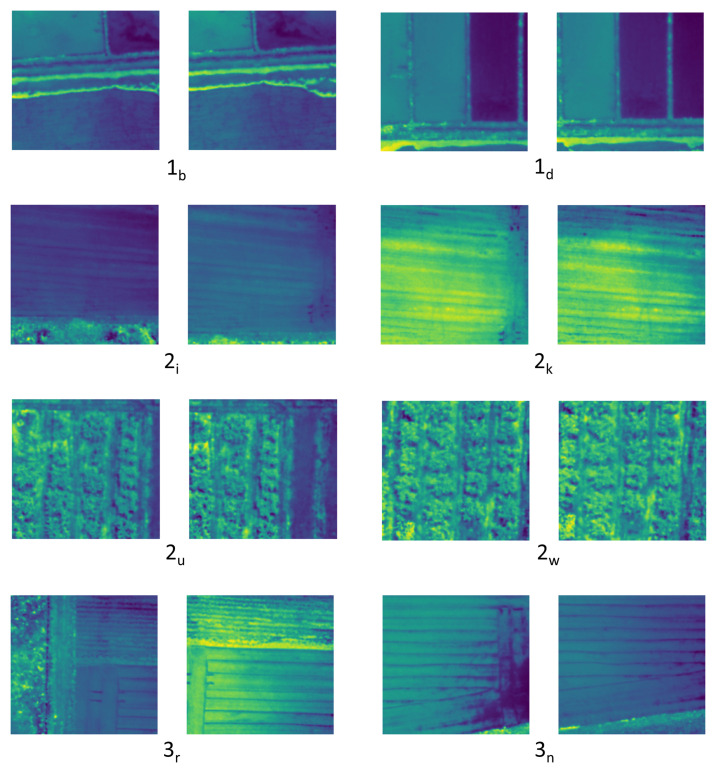
Selected samples from the datasets. Each figure name refers to the corresponding dataset and image pair in Table 1. The dataset number is represented in the base number and subscript presents the image pair.

In order to evaluate the proposed method, four state-of-the-art feature detectors and matches used in Simultaneous Localization and Mapping (SLAM) and other visual tracking tasks were selected, along with the traditional SIFT method. Eighteen image pairs were selected from three data sets taken on different dates and at different locations. The flight altitudes of the three missions were 100 m, 110 m, and 80 m. Samples from each data set are presented in Figure 9, where the main number indicates the dataset number and the subscript indicates the image pair. These image pairs were selected in order to highlight the strengths and weaknesses of the proposed method and compare its results with the state-of-the-art methods. Each image pair was processed through the proposed method as described in Section 3.5 to obtain the SSIM and correlation coefficient. If the identified matches are accurate, they should reflect higher SSIM and correlation values, indicating that the two overlapping regions are similar and that the method successfully produced high-quality feature matches. In addition to the SSIM and correlation coefficient, we obtained the inlier percentage, which falls into the calculated homography.

The obtained results from the evaluation experiments are listed in Table 1. When the match number is present and the rest of the columns indicate “Fail”, this indicates that while the algorithm produced matches, it was unable to calculate a homography using the identified matches or the calculated homography did not produce meaningful results. When the term “Fail” is present without indicating any matches, this implies that the algorithm was not able to produce any matches for the respective image pairs. Overall, the proposed method, LoFTR method, and LightGlue with DISK descriptor–matcher produced far superior performance in terms of the number of matches produced and number of inliers that fell under the calculated homography. With the first dataset, all of the methods were able to produce respectable metrics for the identified feature matches, with the exception of the matches obtained by LoFTR for image pair *f*, which failed to produce any usable homography from the detected matches. In terms of the number of feature matches, traditional SIFT, GFTT + OpenGlue, and KeyNet + OpenGlue produced fewer results compared to the other three methods. This poor performance was far more evident on the second and third datasets; the results when using the above three methods were subpar.

It was observed that most of the evaluated methods performed acceptably when scenes were complex and contained a variety of shapes and pixel intensities, with the exception of the LoFTR method in $2_{u}$, shown in Figure 9. These include scenes such as the ones depicted in $2_{u}$ and $2_{w}$, where complex vegetation is present, or those depicted in $1_{b}$ and $1_{d}$, where the radiometric characteristics of the scene create contrastive pixel regions. This again emphasizes the necessity of developing methods for use in agricultural plots which do not contain such characteristics.

**Figure 10:**
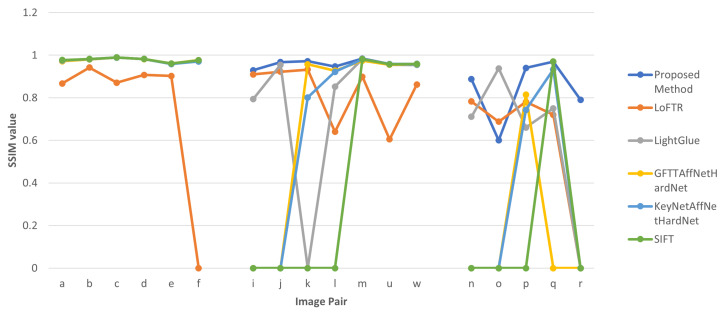
Line graphs showing the SSIM values obtained for each image pair from *a* to *r*.

**Figure 11:**
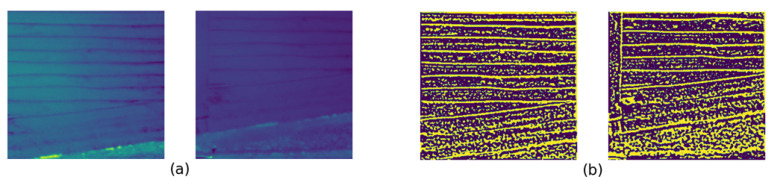
(**a**) Image pair *o* in Dataset 3 and (**b**) the same image pair after the PST edge detection.

Of the evaluated methods, LightGlue produced the highest number of feature matches in most instances. LoFTR was able to produce comparable results with LightGlue; however, it did not perform well for certain image pairs, such as *i* and *r* from the second and third datasets, as shown in Figure 9. The performance of the proposed method in terms of the number of feature matches was between that of LightGlue and LoFTR. Figure 10 presents the SSIM values in graph format. Overall, the proposed method provided the highest SSIM values for the datasets, with the exception of seven instances out of eighteen, five on which SIFT provided the highest SSIM and one each on which LightGlure and GFTT + OpenGlue did so. It should be noted that even though LoFTR provided good results in terms of the number of matches, its results were not as accurate as those of the proposed method or the LightGlue method in terms of SSIM values. Among the feature matches we obtained, the proposed method produced nine of the top eighteen inlier ratios. Additionally, out of the remaining nine cases where our method did not achieve the highest inlier ratio, seven instances were within 10% of the highest value. This solidifies the highly accurate nature of the proposed method. Notably, the proposed method did not perform well on image pair *o*, as depicted in Figure 11. We believe that this is due to two main reasons: first, there is a difference in illumination between the two images, causing the Siamese network to classify the correct matches as non-matches due to the training data mostly consisting of identical pairs from the same image; second, the initial edge-based feature detection method was not able to correctly detect the match because of the low contrast of the image. Finally, it can be observed from the results for each algorithm that the correlation coefficient values are correlated with the SSIM values throughout.

One drawback of the proposed method that needs to be highlighted is its performance speed. Due to the large number of feature matches that need to be filtered out, the proposed algorithm is slower by a factor of ten when compared with the other methods. However, it is important to note that we conducted the experiments while allowing room for further optimization of the code for parallel processing and GPU processing. We propose using the current model as a supplementary feature detection method for use with state-of-the-art methods. For example, in an image mosaicing task the user could incorporate an evaluation metric such as the SSIM to decide whether the detected matches are within a threshold value; if they are not, the proposed method could be utilized to detect the feature matches.

## 5. Conclusions

In this research paper, we propose a novel feature matching method for hyperspectral images. Specifically, we highlight the limitations posed by existing feature detection methods when used with low-resolution hyperspectral images and propose a method involving a 3D convolution-based Siamese network. In the proposed method, a set of feature matches are first generated by performing SIFT feature matching with edge images generated via Phase Stretch Transformation-based edge detection. Then, a Siamese network based on a 3D convolution neural network is used to filter out inaccurate matches, producing a robust set of feature matches. The proposed method factors in all of the information available in every hyperspectral image band, which traditional methods do not take into consideration, in order to produce robust feature matches.

Evaluations carried out using sixteen hyperspectral image pairs revealed that the proposed method can produce highly accurate feature matches, as reflected in high values of the Structural Similarity Index Metric. Although there were a few image pairs for which the proposed method did not produce the highest SSIM results, it was nonetheless competitive with state-of-the-art methods. We believe that the proposed method can aid in the generation of high-quality image mosaics in the remote sensing domain using low pixel resolution hyperspectral images. The same methodology could be used for tasks such as hyperspectral image stitching in other applications as well, such as biomedical research.

A major bottleneck of the proposed method was identified as the initial feature generation step. As part of our future research, we intend to explore ways of refining this method by adopting a different approach to identifying the initial set of feature matches. This alternative strategy is expected to reduce the initial feature set and enhance the performance speed of the proposed method. Furthermore, additional tuning of the Siamese model will be considered in the future to compensate for changes in illumination that might occur due to differences in flight time. We believe that the proposed methodology can aid the advancement of the low-resolution hyperspectral imaging field, thereby making remote sensing accessible for many more researchers. 

## Figures and Tables

**Table 1:** Results obtained with the proposed method.

		**Proposed Method**				**LoFTR**				**LightGlue + DISK**			
**DS**	**IP**	**Matches**	**SSIM**	**Corr**	**Inliers**	**Matches**	**SSIM**	**Corr**	**Inliers**	**Matches**	**SSIM**	**Corr**	**Inliers**
1	a	282	0.973	0.935	94.68	13	0.867	0.851	92.30	677	0.971	0.999	93.83
	b	254	0.980	0.992	91.73	719	0.942	0.978	80.80	834	0.980	0.992	84.77
	c	412	0.989	0.997	95.63	499	0.870	0.964	77.35	838	0.989	0.997	98.56
	**d**	394	**0.982**	0.998	96.70	63	0.907	0.979	85.71	873	0.981	0.998	96.44
	e	132	0.959	0.974	77.20	42	0.902	0.926	92.85	334	0.960	0.974	84.43
	f	231	0.975	0.980	81.38	5	Fail			438	0.973	0.977	98.18
2	**i**	74	**0.929**	0.622	89.18	240	0.909	0.717	87.08	97	0.793	−0.025	82.47
	**j**	88	**0.967**	0.896	87.50	287	0.922	0.833	75.95	387	0.954	0.943	76.74
	**k**	36	**0.971**	0.941	72.22	91	0.932	0.925	69.23	Fail			
	**l**	107	**0.946**	0.983	85.04	7	0.640	0.578	85.71	241	0.853	0.911	68.87
	**m**	215	**0.983**	0.968	86.04	143	0.899	0.880	74.12	212	0.982	0.965	88.21
	**u**	753	**0.958**	0.978	92.69	58	0.605	0.712	93.10	960	0.955	0.976	98.43
	**w**	1057	**0.959**	0.980	99.81	841	0.862	0.929	93.10	1042	0.954	0.977	91.26
3	**n**	14	**0.887**	0.874	100.0	410	0.782	0.747	71.21	6	Fail		
	o	52	0.600	0.693	94.23	234	0.687	0.931	75.21	285	0.710	0.958	92.63
	**p**	131	**0.940**	0.947	94.02	32	0.780	0.514	65.62	507	**0.937**	0.819	81.65
	q	36	0.969	0.980	75.00	45	0.720	0.805	66.66	118	0.660	0.497	33.89
	**r**	23	**0.790**	0.761	91.30	Fail				41	0.750	0.657	60.97
**DS**	**IP**	**GFTTAffNetHardNet + snn**				**KeyNetAffNetHardNet + snn**				**SIFT**			
		**Matches**	**SSIM**	**Corr**	**Inlirs**	**Matches**	**SSIM**	**Corr**	**Inliers**	**Matches**	**SSIM**	**Corr**	**Inliers**
1	a	51	0.971	0.927	98.11	125	0.975	0.982	83.20	38	**0.977**	0.956	13.47
	b	52	0.979	0.992	100.0	126	0.981	0.992	88.09	29	**0.981**	0.993	11.41
	c	87	0.988	0.997	87.35	143	0.989	0.997	95.10	44	**0.989**	0.997	10.67
	d	88	0.981	0.998	95.45	150	0.981	0.998	86.66	38	0.981	0.998	8.12
	e	23	0.960	0.974	86.95	88	0.958	0.974	96.59	33	**0.961**	0.974	18.18
	f	41	**0.976**	0.970	97.56	58	0.970	0.967	89.65	16	0.975	0.980	6.49
2	i	Fail				Fail				34	Fail		
	j	Fail				Fail				Fail			
	k	7	0.957	0.932	100	10	0.801	0.774	80.00	Fail			
	l	4	0.927	0.971	100	18	0.922	0.968	88.88	12	Fail		
	m	27	0.973	0.958	92.59	73	0.981	0.964	84.93	66	0.981	0.963	8.37
	u	192	0.955	0.976	24.70	131	**0.958**	0.978	87.78	357	0.957	0.977	83.75
	w	259	0.958	0.980	24.50	131	0.957	0.979	83.21	456	0.958	0.979	96.49
3	n	Fail				5	Fail			26	Fail		
	o	8	Fail			8	Fail			Fail			
	p	14	0.814	0.510	78.57	41	0.742	0.452	75.60	19	Fail		
	q	1	Fail			26	0.932	0.956	69.23	7	**0.970**	0.978	19.44
	r	1	Fail			6	Fail			Fail			

DS: Dataset. IP: Image Pair. SSIM: Structural Similarity Index Metric. Corr: Correlation Coefficient. Inliers: Values represent percentages.

## Data Availability

Image pairs for testing the proposed method are available via https://github.com/Chamikajp/FeatureDetectionMDPI (accessed on 14 September 2023).

## References

[1] Hasanlou M., Seydi S.T. (2018). Hyperspectral change detection: An experimental comparative study. Int. J. Remote Sens..

[2] Uzkent B., Rangnekar A., Hoffman M. Aerial vehicle tracking by adaptive fusion of hyperspectral likelihood maps. Proceedings of the IEEE Conference on Computer Vision and Pattern Recognition Workshops.

[3] Perera C.J., Premachandra C., Kawanaka H. Feature Detection and Matching for Low-Resolution Hyperspectral Images. Proceedings of the 2023 IEEE International Conference on Consumer Electronics (ICCE) Taiwan.

[4] Sun J., Shen Z., Wang Y., Bao H., Zhou X. LoFTR: Detector-free local feature matching with transformers. Proceedings of the IEEE/CVF Conference on Computer Vision and Pattern Recognition.

[5] Goetz A.F., Vane G., Solomon J.E., Rock B.N. (1985). Imaging spectrometry for earth remote sensing. Science.

[6] Zhong Y., Wang X., Xu Y., Jia T., Cui S., Wei L., Ma A., Zhang L. MINI-UAV borne hyperspectral remote sensing: A review. Proceedings of the 2017 IEEE International Geoscience and Remote Sensing Symposium (IGARSS).

[7] Govender M., Chetty K., Bulcock H. (2007). A review of hyperspectral remote sensing and its application in vegetation and water resource studies. Water SA.

[8] Lu B., Dao P.D., Liu J., He Y., Shang J. (2020). Recent advances of hyperspectral imaging technology and applications in agriculture. Remote Sens..

[9] Yu H., Kong B., Hou Y., Xu X., Chen T., Liu X. (2022). A critical review on applications of hyperspectral remote sensing in crop monitoring. Exp. Agric..

[10] Ghiyamat A., Shafri H.Z. (2010). A review on hyperspectral remote sensing for homogeneous and heterogeneous forest biodiversity assessment. Int. J. Remote Sens..

[11] Wu J., Peng D.L. (2011). Advances in researches on hyperspectral remote sensing forestry information-extracting technology. Spectrosc. Spectr. Anal..

[12] Adam E., Mutanga O., Rugege D. (2010). Multispectral and hyperspectral remote sensing for identification and mapping of wetland vegetation: A review. Wetl. Ecol. Manag..

[13] Veraverbeke S., Dennison P., Gitas I., Hulley G., Kalashnikova O., Katagis T., Kuai L., Meng R., Roberts D., Stavros N. (2018). Hyperspectral remote sensing of fire: State-of-the-art and future perspectives. Remote Sens. Environ..

[14] Shimoni M., Haelterman R., Perneel C. (2019). Hypersectral imaging for military and security applications: Combining myriad processing and sensing techniques. IEEE Geosci. Remote Sens. Mag..

[15] Ramakrishnan D., Bharti R. (2015). Hyperspectral remote sensing and geological applications. Curr. Sci..

[16] Adão T., Hruška J., Pádua L., Bessa J., Peres E., Morais R., Sousa J.J. (2017). Hyperspectral imaging: A review on UAV-based sensors, data processing and applications for agriculture and forestry. Remote Sens..

[17] “GmbH—Real-Time Spectral Imaging”, Cubert. https://www.cubert-hyperspectral.com/products/ultris-5.

[18] XIMEA—Hyperspectral Cameras Based on USB3—xiSpec—ximea.com. https://www.ximea.com/en/products/xilab-application-specific-oem-custom/hyperspectral-cameras-based-on-usb3-xispec.

[19] Datta A., Ghosh S., Ghosh A. (2018). PCA, Kernel PCA and Dimensionality Reduction in Hyperspectral Images. Advances in Principal Component Analysis: Research and Development.

[20] Fabiyi S.D., Murray P., Zabalza J., Ren J. (2021). Folded LDA: Extending the linear discriminant analysis algorithm for feature extraction and data reduction in hyperspectral remote sensing. IEEE J. Sel. Top. Appl. Earth Obs. Remote Sens..

[21] Lennon M., Mercier G., Mouchot M., Hubert-Moy L. Independent component analysis as a tool for the dimensionality reduction and the representation of hyperspectral images. Proceedings of the IGARSS 2001. Scanning the Present and Resolving the Future. IEEE 2001 International Geoscience and Remote Sensing Symposium (Cat. No. 01CH37217).

[22] Fang Y., Li H., Ma Y., Liang K., Hu Y., Zhang S., Wang H. (2014). Dimensionality reduction of hyperspectral images based on robust spatial information using locally linear embedding. IEEE Geosci. Remote Sens. Lett..

[23] Yan L., Niu X. (2014). Spectral-angle-based Laplacian eigenmaps for nonlinear dimensionality reduction of hyperspectral imagery. Photogramm. Eng. Remote Sens..

[24] Ramamurthy M., Robinson Y.H., Vimal S., Suresh A. (2020). Auto encoder based dimensionality reduction and classification using convolutional neural networks for hyperspectral images. Microprocess. Microsyst..

[25] Ayma V., Ayma V., Gutierrez J. (2020). Dimensionality reduction via an orthogonal autoencoder approach for hyperspectral image classification. Int. Arch. Photogramm. Remote Sens. Spat. Inf. Sci..

[26] Pande S., Banerjee B. Dimensionality reduction using 3d residual autoencoder for hyperspectral image classification. Proceedings of the IGARSS 2020—2020 IEEE International Geoscience and Remote Sensing Symposium.

[27] Pande S., Banerjee B. Feedback Convolution Based Autoencoder for Dimensionality Reduction in Hyperspectral Images. Proceedings of the IGARSS 2022-2022 IEEE International Geoscience and Remote Sensing Symposium.

[28] Petersson H., Gustafsson D., Bergstrom D. Hyperspectral image analysis using deep learning—A review. Proceedings of the 2016 Sixth International Conference on Image Processing Theory, Tools and Applications (IPTA).

[29] Kuester J., Gross W., Middelmann W. (2021). 1D-convolutional autoencoder based hyperspectral data compression. Int. Arch. Photogramm. Remote Sens. Spat. Inf. Sci..

[30] Bromley J., Guyon I., LeCun Y., Säckinger E., Shah R. (1993). Signature verification using a “siamese” time delay neural network. Adv. Neural Inf. Process. Syst..

[31] Li Y., Chen C.P., Zhang T. (2022). A survey on siamese network: Methodologies, applications, and opportunities. IEEE Trans. Artif. Intell..

[32] Fu C., Lu K., Zheng G., Ye J., Cao Z., Li B., Lu G. (2022). Siamese object tracking for unmanned aerial vehicle: A review and comprehensive analysis. arXiv.

[33] Wu H., Xu Z., Zhang J., Yan W., Ma X. Face recognition based on convolution siamese networks. Proceedings of the 2017 10th International Congress on Image and Signal Processing, BioMedical Engineering and Informatics (CISP-BMEI).

[34] Xiao W., Ding Y. (2022). A two-stage siamese network model for offline handwritten signature verification. Symmetry.

[35] Jia S., Jiang S., Lin Z., Xu M., Sun W., Huang Q., Zhu J., Jia X. (2021). A semisupervised Siamese network for hyperspectral image classification. IEEE Trans. Geosci. Remote Sens..

[36] Rao W., Gao L., Qu Y., Sun X., Zhang B., Chanussot J. (2022). Siamese transformer network for hyperspectral image target detection. IEEE Trans. Geosci. Remote Sens..

[37] Liu Z., Wang X., Shu M., Li G., Sun C., Liu Z., Zhong Y. An anchor-free Siamese target tracking network for hyperspectral video. Proceedings of the 2021 11th Workshop on Hyperspectral Imaging and Signal Processing: Evolution in Remote Sensing (WHISPERS).

[38] Li Y., Wang J., Yao K. (2022). Modified phase correlation algorithm for image registration based on pyramid. Alex. Eng. J..

[39] Lowe D.G. (2004). Distinctive image features from scale-invariant keypoints. Int. J. Comput. Vis..

[40] Lee H., Lee S., Choi O. (2020). Improved method on image stitching based on optical flow algorithm. Int. J. Eng. Bus. Manag..

[41] Bay H., Ess A., Tuytelaars T., Van Gool L. (2008). Speeded-up robust features (SURF). Comput. Vis. Image Underst..

[42] Harris C., Stephens M. A combined corner and edge detector. Proceedings of the Alvey Vision Conference.

[43] Rosten E., Drummond T. Fusing points and lines for high performance tracking. Proceedings of the Tenth IEEE International Conference on Computer Vision (ICCV’05).

[44] Leutenegger S., Chli M., Siegwart R.Y. BRISK: Binary robust invariant scalable keypoints. Proceedings of the 2011 International Conference on Computer Vision.

[45] Rublee E., Rabaud V., Konolige K., Bradski G. ORB: An efficient alternative to SIFT or SURF. Proceedings of the 2011 International Conference on Computer Vision.

[46] Yi K.M., Trulls E., Lepetit V., Fua P. Lift: Learned invariant feature transform. Proceedings of the Computer Vision–ECCV 2016: 14th European Conference.

[47] DeTone D., Malisiewicz T., Rabinovich A. (2017). Toward geometric deep slam. arXiv.

[48] DeTone D., Malisiewicz T., Rabinovich A. Superpoint: Self-supervised interest point detection and description. Proceedings of the IEEE Conference on Computer Vision and Pattern Recognition Workshops.

[49] Sarlin P.E., DeTone D., Malisiewicz T., Rabinovich A. Superglue: Learning feature matching with graph neural networks. Proceedings of the IEEE/CVF Conference on Computer Vision and Pattern Recognition.

[50] Rocco I., Cimpoi M., Arandjelović R., Torii A., Pajdla T., Sivic J. (2020). Ncnet: Neighbourhood consensus networks for estimating image correspondences. IEEE Trans. Pattern Anal. Mach. Intell..

[51] Rocco I., Arandjelović R., Sivic J. Efficient neighbourhood consensus networks via submanifold sparse convolutions. Proceedings of the Computer Vision–ECCV 2020: 16th European Conference.

[52] Li X., Han K., Li S., Prisacariu V. (2020). Dual-resolution correspondence networks. Adv. Neural Inf. Process. Syst..

[53] Lindenberger P., Sarlin P.E., Pollefeys M. (2023). LightGlue: Local Feature Matching at Light Speed. arXiv.

[54] Yi L., Chen J.M., Zhang G., Xu X., Ming X., Guo W. (2021). Seamless mosaicking of uav-based push-broom hyperspectral images for environment monitoring. Remote Sens..

[55] Peng Z., Ma Y., Mei X., Huang J., Fan F. (2021). Hyperspectral image stitching via optimal seamline detection. IEEE Geosci. Remote Sens. Lett..

[56] Mo Y., Kang X., Duan P., Li S. (2021). A robust UAV hyperspectral image stitching method based on deep feature matching. IEEE Trans. Geosci. Remote Sens..

[57] Zhang Y., Mei X., Ma Y., Jiang X., Peng Z., Huang J. (2022). Hyperspectral Panoramic Image Stitching Using Robust Matching and Adaptive Bundle Adjustment. Remote Sens..

[58] Fang J., Wang X., Zhu T., Liu X., Zhang X., Zhao D. A novel mosaic method for UAV-based hyperspectral images. Proceedings of the IGARSS 2019-2019 IEEE International Geoscience and Remote Sensing Symposium.

[59] Alcantarilla P.F., Bartoli A., Davison A.J. KAZE features. Proceedings of the Computer Vision—ECCV 2012: 12th European Conference on Computer Vision.

[60] Ordóñez Á., Argüello F., Heras D.B. (2018). Alignment of hyperspectral images using KAZE features. Remote Sens..

[61] Li Y., Li Q., Liu Y., Xie W. (2019). A spatial-spectral SIFT for hyperspectral image matching and classification. Pattern Recognit. Lett..

[62] Perera C.J., Premachandra C., Kawanaka H. Comparison of Light Weight Hyperspectral Camera Spectral Signatures with Field Spectral Signatures for Agricultural Applications. Proceedings of the 2023 IEEE International Conference on Consumer Electronics (ICCE).

[63] Asghari M.H., Jalali B. Physics-inspired image edge detection. Proceedings of the 2014 IEEE Global Conference on Signal and Information Processing (GlobalSIP).

[64] Asghari M.H., Jalali B. (2015). Edge detection in digital images using dispersive phase stretch transform. J. Biomed. Imaging.

[65] Coppinger F., Bhushan A., Jalali B. (1999). Photonic time stretch and its application to analog-to-digital conversion. IEEE Trans. Microw. Theory Tech..

[66] Zhou Y., MacPhee C., Suthar M., Jalali B. (2023). PhyCV: The first physics-inspired computer vision library. arXiv.

[67] Canny J. (1986). A computational approach to edge detection. IEEE Trans. Pattern Anal. Mach. Intell..

[68] Sobel I. (2014). History and Definition of the So-Called “Sobel Operator”, More Appropriately Named the Sobel-Feldman Operator. https://www.researchgate.net/publication/239398674_An_Isotropic_3x3_Image_Gradient_Operator.FirstpresentedattheStanfordArtificialIntelligenceProject(SAIL).

[69] Marr D., Hildreth E. (1980). Theory of edge detection. Proc. R. Soc. Lond. Ser. B. Biol. Sci..

[70] Köppen M. The curse of dimensionality. Proceedings of the 5th Online World Conference on Soft Computing in Industrial Applications (WSC5).

[71] Kingma D.P., Ba J. (2014). Adam: A method for stochastic optimization. arXiv.

[72] Bottou L., Curtis F.E., Nocedal J. (2018). Optimization methods for large-scale machine learning. SIAM Rev..

[73] Takase T. (2021). Dynamic batch size tuning based on stopping criterion for neural network training. Neurocomputing.

[74] Wang Z., Bovik A.C., Sheikh H.R., Simoncelli E.P. (2004). Image quality assessment: From error visibility to structural similarity. IEEE Trans. Image Process..

[75] Tyszkiewicz M., Fua P., Trulls E. (2020). DISK: Learning local features with policy gradient. Adv. Neural Inf. Process. Syst..

[76] Shi J. Good features to track. Proceedings of the 1994 IEEE Conference on Computer Vision and Pattern Recognition.

[77] Viniavskyi O., Dobko M., Mishkin D., Dobosevych O. (2022). OpenGlue: Open source graph neural net based pipeline for image matching. arXiv.

[78] Barroso-Laguna A., Riba E., Ponsa D., Mikolajczyk K. Key.Net: Keypoint Detection by Handcrafted and Learned CNN Filters. Proceedings of the IEEE/CVF International Conference on Computer Vision (ICCV).

